# The biological role of metabolic reprogramming in pancreatic cancer

**DOI:** 10.1002/mco2.37

**Published:** 2020-11-05

**Authors:** Tatsunori Suzuki, Motoyuki Otsuka, Takahiro Seimiya, Takuma Iwata, Takahiro Kishikawa, Kazuhiko Koike

**Affiliations:** ^1^ Department of Gastroenterology Graduate School of Medicine The University of Tokyo Tokyo Japan

**Keywords:** autophagy, macropinocytosis, metabolism, pancreatic cancer, tumor microenvironment

## Abstract

Pancreatic ductal adenocarcinoma (PDAC) is a lethal disease and highly resistant to all forms of therapy. PDAC cells reprogram their metabolism extensively to promote their survival and growth. Reflecting the vital role of altered metabolism, experimental and clinical trials targeting the rewired metabolism are currently underway. In this review, we summarize the vital role of metabolic reprogramming in the development of PDAC and the future of novel therapeutic applications.

## Graphical abstract

Various types of metabolic reprogramming occur in pancreatic cancer cells, including metabolism of glucose, amino acids, and nutrient acquisition such as autophagy. Increased knowledge of these features can lead to the development of novel therapeutics.

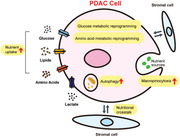

## 1 INTRODUCTION

Pancreatic ductal adenocarcinoma (PDAC) is one of the most lethal cancers, characterized by early metastasis and resistance to all forms of treatment.^1^, ^2^ The extremely high mortality rate of patients with PDAC can be attributed to the lack of both an early diagnosis and appropriate targeted therapy.^3^ Because the pancreas is located in a place difficult to observe, the early diagnosis of PDAC in routine examinations is nearly impossible.^4^ Furthermore, current biomarkers are inadequate to detect PDAC efficiently, especially in the early stages.^5^

In addition to the diagnostic shortcomings, effective therapeutic options are limited. Although improvements in chemotherapy have been achieved with the emergence of new combination chemotherapies,^6^, ^7^ the rapid development of chemoresistance usually leads to poor prognosis. Therefore, new treatment strategies are urgently required to improve the prognosis.

Recently, metabolic reprogramming, an emerging hallmark of cancer,^8^ has generated renewed interest. Cancer cells rewire their metabolism to promote their growth and proliferation.^9^ Based on recent evidence of metabolic adaptation in PDAC cells,^10^, ^11^, ^12^, ^13^, ^14^ the metabolic features of PDAC could constitute attractive therapeutic opportunities.

In this review, we discuss how PDAC cells alter their metabolism to facilitate growth and how metabolism‐targeted therapies could be used to improve the prognosis of patients with PDAC.

## 2 GLUCOSE METABOLISM

Glucose is a major nutrient in cellular metabolism and biosynthesis. When used as a nutrient in normal cells, glucose is converted into carbon dioxide in mitochondria via oxidative phosphorylation to produce ATP. By contrast, cancer cells utilize more glucose carbon for anabolic reactions such as synthesis of ribose, amino acids, lipids, and glycosylation precursors.^15^

**FIGURE 1:**
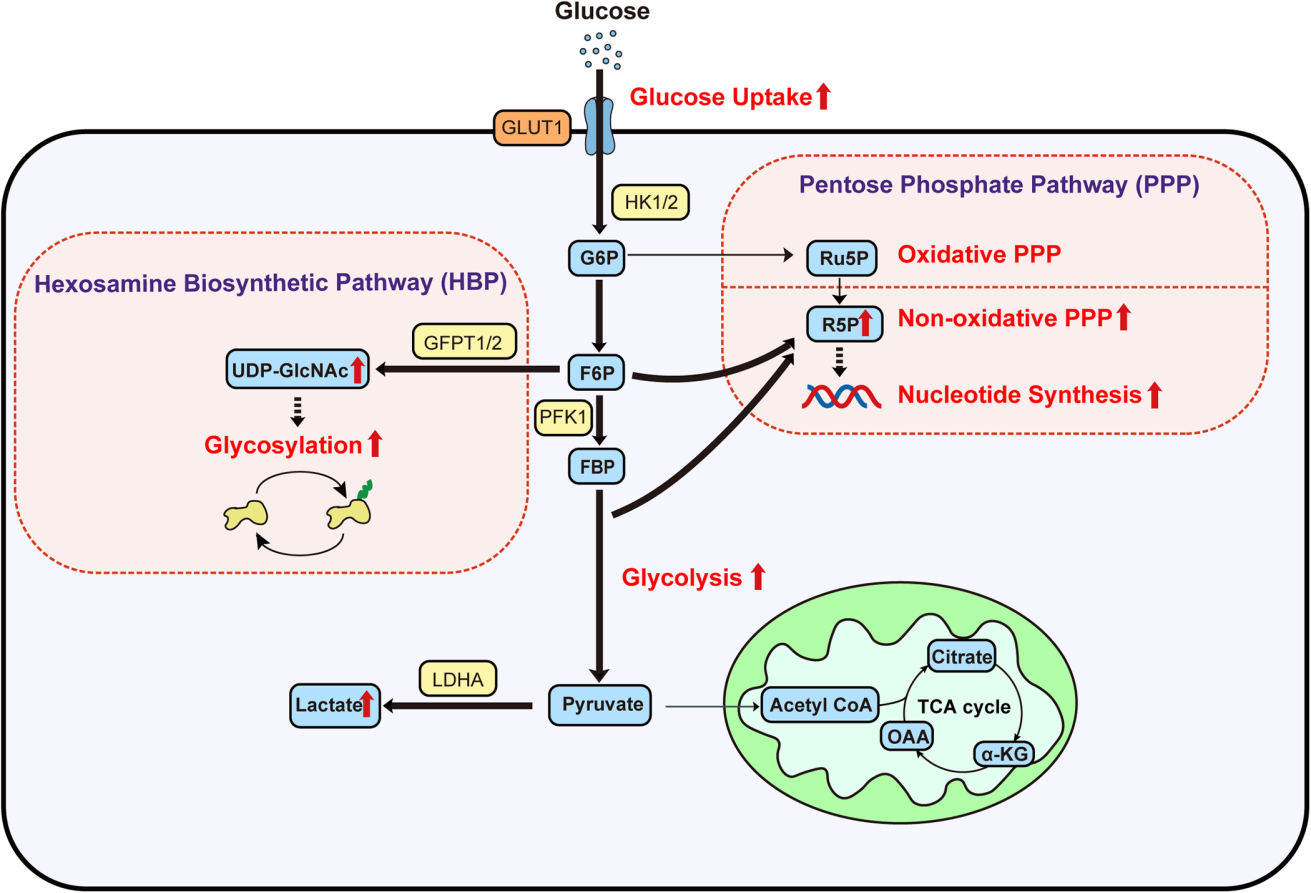
Glucose metabolism in PDAC cells. PDAC cells increase glucose uptake through oncogenic KRAS‐mediated upregulation of GLUT1. Oncogenic KRAS also upregulates other glycolytic enzymes, resulting in increased glycolytic flux. Glucose carbon is also important for anabolic metabolism in the HBP and the nonoxidative PPP phase. Bold arrows indicate accelerated metabolic pathways. α‐KG, α‐ketoglutarate; FBP, fructose 1,6‐bisphosphate; F6P, fructose 6‐phosphate; G6P, glucose 6‐phosphate; GLUT1, glucose transporter 1; GFPT1/2, glutamine fructose 6‐phosphate transamidase 1/2; HBP, hexosamine biosynthetic pathway; HK1/2, hexokinase 1/2; LDHA, lactate dehydrogenase A; OAA, oxaloacetate; PDAC, pancreatic ductal adenocarcinoma; PFK1, phosphofructokinase 1; R5P, ribose 5‐phosphate; Ru5P, ribulose 5‐phosphate; UDP‐GlcNAc, UDP‐*N*‐acetylglucosamine

In PDAC cells, oncogenic KRAS upregulates glucose transporter 1 (GLUT1), which increases glucose uptake^10^, ^16^ (Figure 1). Oncogenic KRAS also upregulates hexokinase 1/2 (HK1/2), phosphofructokinase 1, and lactate dehydrogenase A (LDHA) to promote glycolysis.^10^, ^17^ Furthermore, the hypoxic microenvironment and other mechanisms cooperate with oncogenic KRAS to increase the expression of glycolytic enzymes and maintain cytosolic ATP.^18^, ^19^, ^20^, ^21^ In addition to the transcriptional upregulation of glucose transporters and glycolytic enzymes, KRAS4A interacts with HK1 in mitochondria and regulates HK1 directly.^22^

Glucose also plays a crucial role in the anabolic pathway. Oncogenic KRAS activates the hexosamine biosynthetic pathway (HBP),^10^, ^23^ producing uridine diphosphate‐*N*‐acetylglucosamine, which has numerous functions, including intracellular signaling and posttranslational modification.^24^ Oncogenic KRAS increases the HBP flux via the transcriptional upregulation of glutamine fructose 6‐phosphate transamidase 1 (GFPT1).^10^ HBP flux is also increased through GFPT2, which is induced by hypoxia.^25^

The pentose phosphate pathway (PPP) is another anabolic pathway through which oncogenic KRAS increases the glucose flux. This pathway is important for producing nucleotide synthesis intermediates and is subdivided into the oxidative and nonoxidative phases. In the oxidative phase, glucose 6‐phospate is converted into ribulose 5‐phosphate and two molecules of NADPH are produced simultaneously. Then, NADPH is used for redox control and fatty acid synthesis. The nonoxidative PPP phase consists of reactions that produce ribose 5‐phophate (R5P) for nucleotide synthesis. In a previous study, oncogenic KRAS in PDAC cells became dependent on the nonoxidative PPP phase^10^ through MYC upregulation.^26^ MUC1 also helps induce anabolic glucose metabolism by stabilizing HIF1α.^27^, ^28^, ^29^, ^30^ Because normal cells produce R5P in the oxidative phase, this differential reliance on the nonoxidative phase could be a metabolic vulnerability of pancreatic cancer. These glycolytic changes begin at the time of precancerous lesions and are maintained during tumor progression.^31^

## 3 AMINO ACID METABOLISM

**FIGURE 2:**
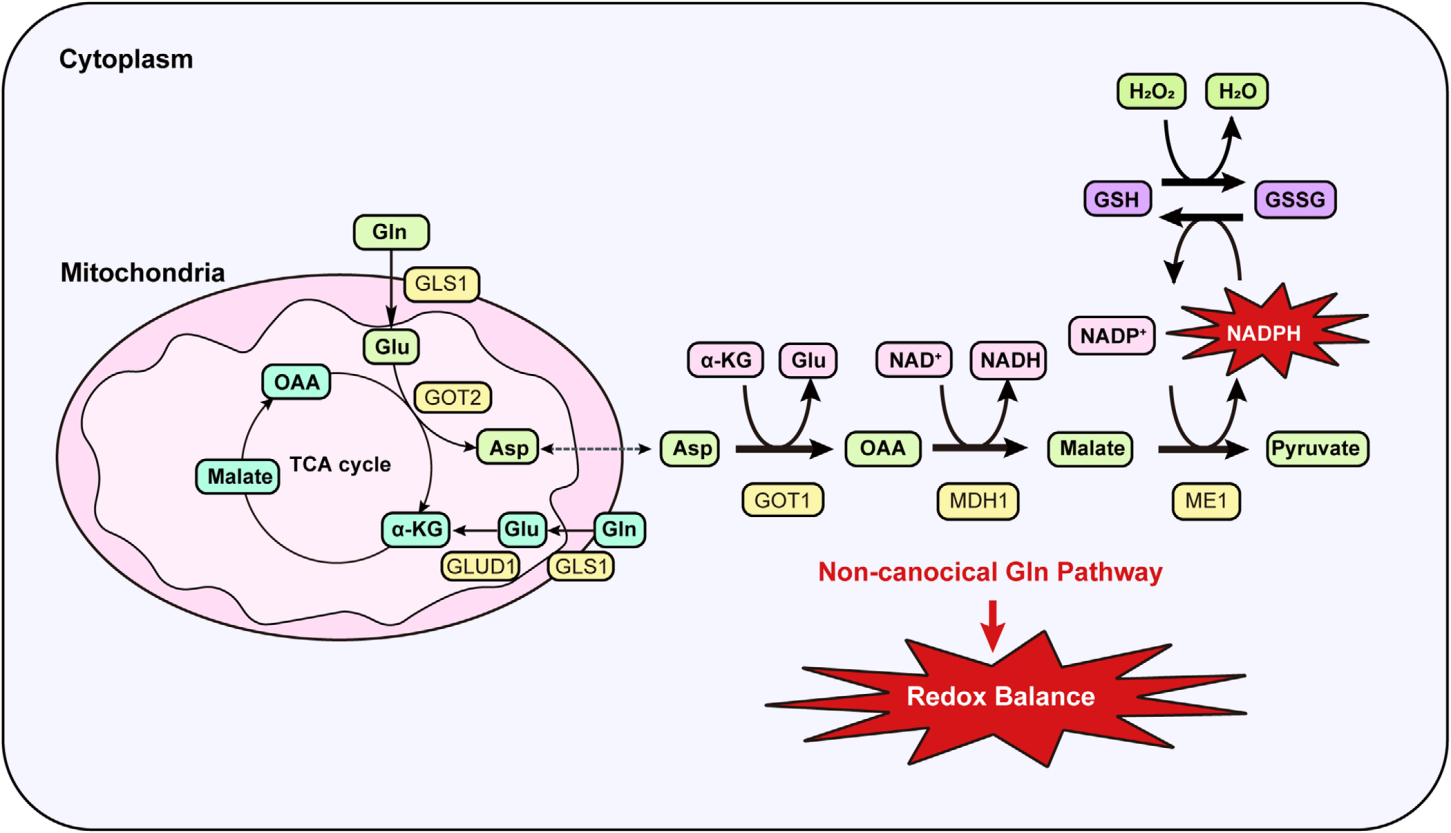
Glutamine metabolism in PDAC cells. PDAC cells depend on the noncanonical Gln pathway for redox balance. Gln‐derived Glu is metabolized to Asp by GOT2. This Asp is transported into the cytoplasm and then metabolized to OAA by GOT1. This OAA is metabolized to malate and then pyruvate, increasing the NADPH/NADP^+^ ratio. This sustains the reduced GSH levels needed for redox balance. α‐KG, α‐ketoglutarate; Asp, aspartate; GOT1, aspartate aminotransferase 1; GOT2, aspartate aminotransferase 2; Glu, glutamate; GLUD1, glutamate dehydrogenase 1; GLS1, glutaminase 1; Gln, glutamine; GSH, glutathione; MDH1, malate dehydrogenase 1; ME1, malic enzyme 1; OAA, oxaloacetate; GSSG, oxidized glutathione; PDAC, pancreatic ductal adenocarcinoma

PDAC cells also reprogram amino acid metabolism. Various amino acid transporters are upregulated markedly in PDAC cells to meet the metabolic demand.^32^, ^33^ The amino acid glutamine (Gln) is important for tumor cell as a major source of carbon and nitrogen, contributing to biosynthesis of macromolecules^34^ (Figure 2).

Gln‐derived carbon continuously replenishes the tricarboxylic acid (TCA) cycle to produce reducing equivalents and intermediates for macromolecular synthesis. In the canonical Gln pathway, Gln‐derived glutamate (Glu) is converted into α‐ketoglutarate (α‐KG), replenishing the TCA cycle. In this process, NADH and the precursors of macromolecules and lipids are produced. Gln is also an important source of purines and pyrimidines.^35^, ^36^ Gln itself and Gln‐derived aspartate (Asp) can be used as substrates for nucleotide synthesis.^37^

In addition, PDAC cells utilize Gln to maintain redox homeostasis.^11^ Gln plays two roles in this process. First, Gln is a resource for the synthesis of glutathione, a tripeptide (composed of Glu, cysteine, and glycine) that protects cells from free radical damage by acting as an antioxidant. Second, oncogenic KRAS promotes the production of reducing equivalents in the form of NADPH via a noncanonical Gln metabolism pathway. Gln‐derived Glu is converted into Asp by aspartate aminotransferase (GOT2) in mitochondria. This Asp is transported into the cytoplasm and then metabolized by cytosolic aspartate aminotransferase (GOT1), malate dehydrogenase, and malic enzyme (ME1), which results in the production of reducing potential in the form of NADPH. GOT1 also plays an important role in an acidic tumor microenvironment.^38^ PDAC cells can maintain the redox balance under acidosis stress by increasing anaplerotic Gln metabolism.

In addition to the role of KRAS in metabolic changes, p53 also plays an important role in rewiring Gln and glucose metabolism to accumulate α‐KG.^39^ Accumulated α‐KG undergoes chromatin modification and exerts a p53‐mediated tumor suppressor effect.

The metabolism of other amino acids is reprogrammed in PDAC cells. Proline derived from the extracellular matrix (ECM) promotes PDAC cell survival under nutrient‐limited conditions.^40^ Through macropinocytosis‐dependent and macropinocytosis‐independent mechanisms, PDAC cells take up ECM collagens to replenish TCA cycle when other fuels are limited. Cysteine is also important for supporting PDAC cell survival through the maintenance of nutritional and oxidative homeostasis.^41^ The cystine/glutamate exchanger xCT is important for maintaining cysteine balance and may be a promising therapeutic target. Furthermore, elevation of plasma branched‐chain amino acids is associated with a future diagnosis of PDAC, which might be due to increased breakdown of tissue protein.^42^, ^43^

## 4 LIPID METABOLISM

Lipid metabolism is also important for PDAC progression.^44^ In PDAC cells, cholesterol uptake and many enzymes associated with fatty acids and cholesterol synthesis are significantly upregulated.^45^, ^46^ A recent study showed that sterol O‐acyltransferase 1 (SOAT1) promotes the mevalonate pathway, preventing cholesterol feedback inhibition by unesterified cholesterol in PDAC cells with a p53 mutation and loss of heterozygosity.^47^ By contrast, inhibition of SOAT1 does not affect the growth of normal cells with wild‐type p53, suggesting a potential therapeutic window.

## 5 NUTRIENT ACQUISITION

**FIGURE 3:**
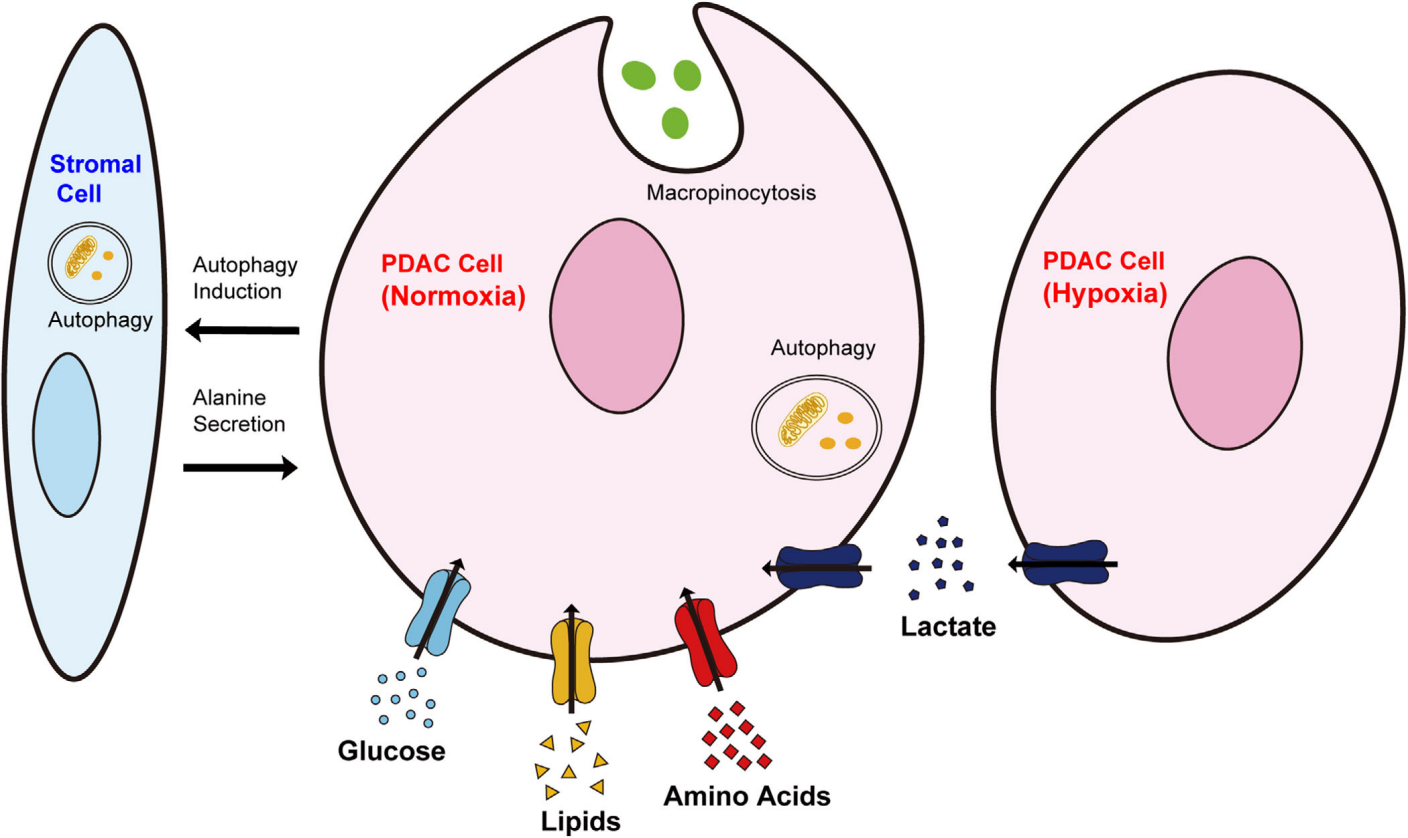
Nutrient acquisition strategies utilized by PDAC cells. PDAC cells acquire nutrients in various ways. The uptake of glucose, amino acids, and lipids is enhanced in PDAC cells. Macropinocytosis and autophagy are also promoted. PDAC cells undergo metabolic crosstalk with stromal cells. PDAC cells stimulate autophagy in stromal cells, inducing alanine secretion. Metabolic crosstalk also occurs among cancer cells. Lactate secreted by PDAC cells in the hypoxic region is taken up by PDAC cells in the normoxic region. These mechanisms cooperate to promote PDAC growth. PDAC, pancreatic ductal adenocarcinoma

PDAC cells are poorly vascularized and in a state of nutrient deprivation.^48^, ^49^ Thus, PDAC cells have alternative mechanisms by which they acquire nutrients needed to survive and grow. To acquire sufficient fuel, PDAC cells activate mechanisms, such as autophagy, macropinocytosis, and metabolic interaction with surrounding noncancerous cells within the tumor microenvironment (Figure 3).

### 5.1 Autophagy

Autophagy is a degradative process involving the formation of autophagosomes that swallow intracellular components for delivery to lysosomes.^50^ The engulfed components are degraded by fusion of the autophagosome and lysosome, and the digested biomolecules are recycled as cellular nutrients^51^ (Figure 3). Autophagy is regulated by highly controlled signaling events occurring at a basal level and is triggered by diverse signals.^52^

In the progression of PDAC, autophagy plays critical but opposing roles.^53^ At the tumor initiation stage, autophagy can be tumor suppressive via cellular quality control mechanism. In an established tumor, autophagy can be tumor promoting via an intracellular recycling mechanism, indicating that autophagy is essential for PDAC tumor growth.^54^ The inhibition of autophagy in genetically engineered mouse models suppressed the progression of PDAC.^55^, ^56^, ^57^

The autophagic reliance of PDAC was recently shown to be partly mediated by MiT/TFE family of transcriptional factors.^58^ In PDAC, microphthalmia/transcription factor E (MiT/TFE) proteins can activate basal autophagy independently of mechanistic target of rapamycin (mTOR) activity and are necessary for the maintenance of amino acid pools. Furthermore, an ULK1 phosphatase (PP2A‐B55α complex), which activates autophagy, was also shown to be crucial for high levels of basal autophagy in PDAC.^59^

### 5.2 Macropinocytosis

Although autophagy plays an important role in metabolic scavenging, it cannot create a net increase in biomass. PDAC cells are also dependent on another pathway to satisfy their metabolic needs. Macropinocytosis is an endocytic process that involves the nonspecific uptake of extracellular material through large, heterogeneous vesicles known as macropinosomes.^60^ The internalized molecules undergo lysosomal degradation, yielding precursor molecules that can be used for macromolecular biosynthesis.

In PDAC cells, oncogenic KRAS promotes macropinocytosis to acquire protein sources to survive in nutrient‐poor conditions by replenishing amino acid pools^48^, ^49^, ^61^, ^62^, ^63^, ^64^ (Figure 3). Enhanced macropinocytosis might enhance the delivery of nanomedicines, such as nanoparticle albumin‐bound (nab)‐paclitaxel, and partly explain the efficacy of the drug.^7^, ^65^

### 5.3 Metabolic crosstalk with the microenvironment

The molecular and cellular heterogeneity of PDAC is well characterized.^66^, ^67^, ^68^, ^69^ Metabolic heterogeneity also exists in cancer cells,^9^, ^70^, ^71^, ^72^ which depends on differences in cell state. For instance, pancreatic cancer stem cells are mainly dependent on oxidative phosphorylation, but have metabolic plasticity leading to resistance of mitochondrial inhibition.^73^ MYC and PGC‐1α cooperatively determine such plasticity. In addition, environmental factors, such as local nutrient and oxygen status, affect metabolic heterogeneity.

Accordingly, metabolically different cell populations utilize cross‐feeding mechanisms in which one population can use metabolites from another (Figure 3). A recent study showed that cancer cells were shown to use lactate as a substrate for the TCA cycle.^74^ Lactate secreted by cancer cells in the hypoxic region is captured by cancer cells in the normoxic region and feeds cancer cells.^25^ PDAC cells upregulate the expression of monocarboxylate transporter 1 (MCT1) and MCT4 to promote lactate exchange.^75^, ^76^ Lactate secreted by cancer cells is also captured by mesenchymal stem cells and converted into α‐KG, causing extensive epigenetic changes and promoting cancer‐associated fibroblast differentiation.^77^

Metabolites derived from stromal cells also feed cancer cells. A recent study showed that stroma‐associated pancreatic stellate cells (PSCs) had an important role in PDAC proliferation via the release of free amino acids, especially alanine.^78^ PDAC cells activate autophagy in PSCs and selectively utilize the released alanine, fueling the TCA cycle.

### 5.4 Preclinical and clinical trials targeting cancer metabolism and conclusion

Although metabolism‐targeted therapy is not yet standard therapy for many cancers, several experimental and clinical trials targeting altered metabolism are currently underway.

In vitro and in preclinical mouse models, WZB117, a specific GLUT1 inhibitor, was shown to be a promising agent targeting cancer stem cells.^79^ In preclinical mouse models, FX11, a LDHA inhibitor, was also shown to suppress tumor growth.^80^, ^81^ In a phase I clinical trial, 2‐deoxy‐D‐glucose in combination with docetaxel was shown to be feasible with clinical benefit (NCT00096707).^82^ For mitochondrial metabolism, the mitochondrial inhibitor phenformin showed antitumor effects in PDAC xenograft models.^83^ For altered Gln metabolism, GLS1 inhibitors and β‐lapachone (ARQ761) selectively induced PDAC cell death in vivo.^84^ A phase I clinical trial using ARQ761 in combination with gemcitabine/nab‐paclitaxel in PDAC is currently underway (NCT02514031). For lipid metabolism, SB‐204990, an ATP citrate lyase inhibitor, reduced tumor growth in vivo.^85^

In addition, the autophagy inhibitor hydroxychloroquine is under clinical evaluation. Hydroxychloroquine alone has shown limited efficacy,^86^, ^87^ but was clinically beneficial in combination with gemcitabine/nab‐paclitaxel (NCT01978184). In a recent study, dual inhibition of the RAF‐MEK‐ERK pathway and autophagy synergistically impaired PDAC proliferation.^88^, ^89^ Because direct RAS inhibitors, previously thought to be undruggable, and more specific autophagy inhibitors are under development; their use could potentially enhance the therapeutic effect.^90^, ^91^

Although attempts to treat PDAC by targeting tumor metabolism have succeeded to some extent, the development of resistance is a concern when designing metabolism‐targeted therapy. Metabolic networks are very plastic and reportedly can be rewired to avoid targeted therapies.^92^, ^93^ In addition, PDAC cells have heterogeneous metabolic subtypes.^70^, ^71^ Different metabolic subtypes have different prognoses and metabolic vulnerability.^94^, ^95^ Therefore, these factors should be considered when designing effective metabolism‐targeted therapy. In the near future, novel treatment strategies based on the findings of PDAC metabolism are expected to improve the prognosis of patients with PDAC.

## CONFLICT OF INTEREST

The authors declare no conflict of interest.

## AUTHOR CONTRIBUTIONS

TS, MO, and KK wrote the manuscript. TS, TI, and TK prepared the figures.
